# Comments on Cultured Human Sarcoma Cells

**DOI:** 10.1080/13577140310001607301

**Published:** 2003-06

**Authors:** Joseph G. Sinkovics

**Affiliations:** Cancer Institute St. Joseph's Hospital Departments of Medical Microbiology-Immunology and Medicine The University of South Florida College of Medicine & The H.L. Moffitt Cancer Center Tampa FL 33612 USA

## Abstract

Human sarcoma cell populations maintained in culture reflect to the native tumor cells better if the culture retains those nonmalignant cells that comprised the tumor's microenvironment *in vivo* [Hu M, *et al*. Characterization of 11 human sarcoma cell strains. *Cancer* 2002; 95: 1569–76] and thus provide paracrine growth factors and protection from apoptotic death to the tumor cells. Whereas sarcoma cell cultures obtained through meticulous efforts aimed at the elimination of all non-malignant cells of the tumor's original microenvironment consist of subpopulations of tumor cells growing exclusively with the support of their own autocrine growth loops [Sinkovics JG, *et al*. Growth of human tumor cells in established cultures. In: Busch H, ed. *Methods in Cancer Research*. Vol 14. New York: Academic Press, 1978; 243–323].


*Sarcoma*, June 2003, *VOL. 7, NO. 2, 75–77*

SHORT COMMUNICATION

### Comments on cultured human sarcoma cells

JOSEPH G. SINKOVICS

*Cancer Institute, St. Joseph's Hospital, Departments of Medical Microbiology-Immunology and Medicine, The University of South Florida College of Medicine & The H.L. Moffitt Cancer Center, Tampa, FL 33612, USA*

**Abstract**
Human sarcoma cell populations maintained in culture reflect to the native tumor cells better if the culture retains those nonmalignant cells that comprised the tumor's microenvironment *in vivo* [Hu M, *et al.* Characterization of 11 human sarcoma cell strains. *Cancer* 2002; 95: 1569–76] and thus provide paracrine growth factors and protection from apoptotic death to the tumor cells. Whereas sarcoma cell cultures obtained through meticulous efforts aimed at the elimination of all non-malignant cells of the tumor's original microenvironment consist of subpopulations of tumor cells growing exclusively with the support of their own autocrine growth loops [Sinkovics JG, *et al.* Growth of human tumor cells in established cultures. In: Busch H, ed. *Methods in Cancer Research*. Vol 14. New York: Academic Press, 1978; 243–323].

It has been some 30 years since that, at the Section of Clinical Tumor Virology and Immunology, Department of Medicine, The University of Texas M.D. Anderson Hospital, we established over 50 human tumor cell lines,[1] among them 25 deriving from sarcomas.

One purpose of the study was a search for causative retroviruses, since from the chicken through mice up to some simian species causative sarcoma viruses were isolated and identified. Filtered supernatant fluids of human sarcoma cell cultures occasionally caused 'antigenic conversion' (as shown by immunofluorescence stains) and 'focus formation' in cultures of human embryonic fibroblasts.[2] These phenomena could not be propagated by passages and yielded no human sarcoma viral isolates, even though transmission EM pictures showed rare retroviral particles in some of these cells.[3] Some xenotropic murine retroviral particles showed up in cultured human sarcoma cells xenografted into nude mice.[1] Another purpose of the study was to test autologous and allogeneic patient lymphocytes for cytotoxicity against cultured sarcoma cells and correlate these reactions with the clinical course of the patients.[4,5] Some spectacular interaction between sarcoma cells and lymphocytes occurred and were photographed. Allogeneic large lymphocytes with granular cytoplasm lysed sarcoma cells; smaller autologous lymphocytes with thinner rim of cytoplasm and compact nucleus caused clumping in the nuclei of sarcoma cells.[6–8] These purely morphological observations gained their explanations later when NK cells and their derivatives (LAK cells) with their receptors recognizing malignantly transformed cells (KIRs; NKp30,44,46; NKG2D) and subtypes of immune T cells (TIL) were separated; and perforins of lymphocyte origin and death ligands and their receptors (TNF$\alpha$; FasL$\rightarrow$ FasR CD95) inducing extrinsic apoptosis were discovered elsewhere. Furthermore nuclear clumping and death of the lymphocytes attacking sarcoma cells also occurred: the 'counterattack on lymphocytes', while sarcoma cells remained intact[8,9] (Fig. 1); these pictures were taken long before the discovery of the Fas system. A third and main purpose of these studies was the preparation of 'viral oncolysates' from some of these cell lines for active tumor-specific immunotherapy against sarcomas[10] and melanoma.[11,12]

Our work as reported biannually in the institutional *Research Report* from 1960 to 1978 preceded the discovery of oncogenes whereby a retrovirus transduces a cellular protooncogene (for example c-*myc*, c-*sis*, c-*src*, etc.) and thus gains oncogenicity;[13] and it was carried out in the absence of the as yet undiscovered monoclonal antibodies[14] for lymphocyte subtyping. Decades have since gone by and we still do not have an established human sarcoma vaccine to prevent potsurgical relapses and we do not effectively utilize immune lymphocytes for adoptive immunotherapy of metastatic disease.

We now know that our aim at establishing human tumor cell lines was misdirected. When we forcefully and with great efforts separated tumor cells from normal connective tissue cells that accompanied them and changed media diligently, even on weekends, we deprived our cultures of the as yet undiscovered paracrine growth factors and thus selected

ISSN 1357-714X print/1369-1643 © 2003 Taylor & Francis Ltd
DOI: 10.1080/13577140310001607301

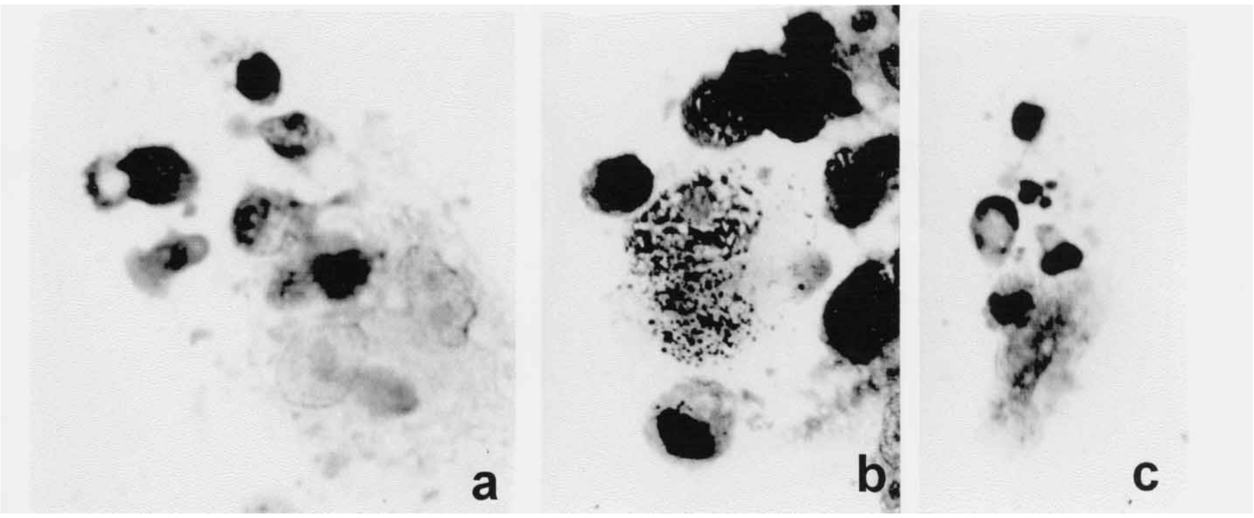

**Fig. 1.** *(a) Allogeneic large lymphocytes lyse the cytoplasm of two sarcoma cells. (b) Two autologous lymphocytes kill two melanoma cells by causing nuclear clumping. (c) Autologous lymphocytes attacking fibrosarcoma cell undergo nuclear clumping and die while sarcoma cell survives. J.G. Sinkovics, M.D. Anderson Hospital, Houston TX (1972–1974).*

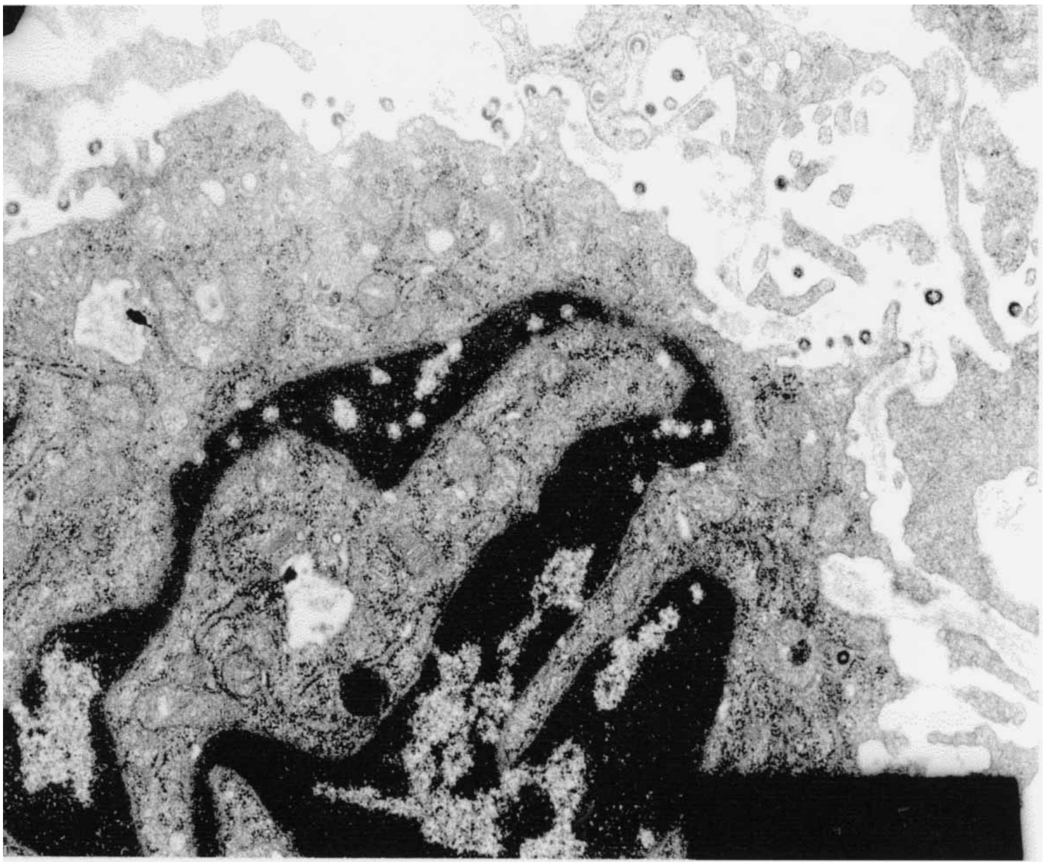

**Fig. 2.** *Kaposi sarcoma cell with budding retroviral particles that failed to react with commercial kits aimed at HTLV-I or HIV-1 structural proteins. F. Gyorkey† and J.G. Sinkovics, VA Medical Center, Houston TX, 1983 (unpublished).*

out subpopulations of those tumor cells that were growing with the support of their own autocrine growth loops.

It was especially difficult to establish cell lines from classical pre-AIDS Kaposi's sarcoma (KS) tumors: we continued to refresh the media of their primary cultures sometimes daily and did not use 'conditioned media'. In addition to herpesviruses (CMV; EBV in the B-lineage lymphocytes infiltrating the tumor; and now HHV-8), KS cells occasionally displayed budding retrovirus particles[15,16] (Fig. 2). We are now promoting a new working hypothesis that a retroviral dsDNA provirus deprived of *env* gene (but occasionally capable of acquiring one) exists in these cells inserted next to the *int*-bFGF gene family, which is thus transactivated and amplified to encode bFGF. When excess bFGF ligands are released and captured by their receptors as expressed by the same cells, an autocrine growth loop is established.

The recent outstanding report[17] from the same institution recognizes the interaction whereby subverted stromal cells serve the tumor cells with growth factors of paracrine origin. This is a highly generalized phenomenon[12] and it is applicable to many tumors other than sarcomas. For example, certain melanoma cells use the Fas ligand (L) as an autocrine-paracrine growth factor;[18] neuroblastoma cells are driven to grow by TNF$\alpha$;[19] lymphoma cells armed with FasL kill host immune T cells;[20] and CLL cells are protected from apoptosis by IFN$\gamma$ or by stromal cell-derived factor-1 (SDF-1) released from subverted fibroblast-like cells of the host.[20] While the c-*sis*-product PDGF and molecular mediators of neoangiogenesis (bFGF; VEGF and their receptors) are well recognized growth factors for sarcoma cells, SDF-1 has not as yet received the attention it deserves.

### References

1. Sinkovics JG, Györkey F, Kusyk C, Siciliano MJ. Growth of human tumor cells in established cultures. In: Busch H, ed. *Methods in Cancer Research*. Vol 14. New York: Academic Press, 1978; 243–323.
2. Sinkovics JG, Plager C, McMurtrey MJ, *et al.* Immunotherapy of human sarcomas. In: *Primary Management of Bone and Soft Tissue Tumors*. Twenty-first Annual Clinical Conference on Cancer, 1976, at The University of Texas System Cancer Center M.D. Anderson Hospital and Tumor Institute, Houston, TX. Chicago, IL: Year Book Medical Publishers, Inc. 1977; 361–410.
3. Gyorkey F, Sinkovics JG, Gyorkey P. Electron microscopic observations on structures resembling myxovirus in human sarcomas. *Cancer* 1971; 27: 1449–54.
4. Sinkovics JG, Kay HD, Thota H. The evaluation of chemoimmunotherapy regimens by in vitro lymphocyte cytotoxicity directed to cultured human tumor cells. In: *Bibliotheca Haematologica*. Vol 43. Basel, New York: S. Karger, 1976; 281–4.
5. Sinkovics JG. Complete remissions lasting over three years in adult patients treated for metastatic sarcoma. In: Crispen RG, ed. *Tumor Progression*. Amsterdam, New York: Elsevier North-Holland, 1980; 315–31.
6. Sinkovics JG. A reappraisal of cytotoxic lymphocytes in human tumor immunology. In: Cory JG, Szentivanyi A, ed. *Cancer Biology and Therapeutics*. The First Annual H. Lee Moffitt International Symposium. New York, London: Plenum Press, 1987; 225–53.
7. Sinkovics JG. Cytotoxic lymphocytes. *Ann Clin Lab Sci* 1986; 16: 488–96.
8. Sinkovics JG. Programmed cell death (apoptosis): its virological and immunological connections. *Acta Microbiol Hung* 1991; 38: 321–34.
9. Sinkovics JG. Malignant lymphoma arising from natural killer cells: report of the first case in 1970 and newer developments in the FasL→FasR (CD95) system. *Acta Microbiol Immunol Hung* 1997; 44: 295–307.
10. Sinkovics JG, Plager C, Romero JJ. Immunology and immunotherapy of patients with sarcomas. In: Crispen RG, ed. *Immunotherapy of Solid Tumors*. Philadelphia, PA: Franklin Institute Press, 1977; 211–9.
11. Sinkovics JG, Plager C, McMurtrey M, Papadopoulos NE, *et al.* Adjuvant chemoimmunotherapy for malignant melanoma. In: Crispen RG, ed. *Neoplasm Immunity: Experimental and Clinical*. Amsterdam, Oxford: Elsevier North-Holland, 1980; 481–519.
12. Sinkovics JG, Horvath JC. Vaccination against human cancers. *Int J Oncol* 2000; 16: 81–96.
13. Sinkovics JG. Oncogenes and growth factors. *Crit Rev Immunol* 1988; 8: 217–98.
14. Sinkovics JG, Dreesman G. Monoclonal antibodies of hybridomas. *Rev Infect Dis* 1983; 5: 9–34.
15. Gyorkey F, Sinkovics JG, Melnick JL, Gyorkey P. Retroviruses in Kaposi sarcoma cells in AIDS. *New Engl J Med* 1984; 311: 1183–4.
16. Sinkovics JG, Horvath JC. Kaposi's sarcoma: breeding ground of Herpesviridae. A tour de force over viral evolution. *Int J Oncol* 1999; 14: 615–46.
17. Hu M, Nicolson GL, Yu D, *et al.* Characterization of 11 human sarcoma cell strains. *Cancer* 2002; 95: 1569–76.
18. Horvath JC, Horvath E, Sinkovics JG, *et al.* Human melanoma cells (HMC) eliminate autologous host lymphocytes ($Ly^{FasR+}$) and escape apoptotic death (A) by utilizing the FasL→FasR system as an autocrine growth loop ($HMC^{FasL+FasR+}$). 89th Annual Meeting. *Proc Am Assoc Cancer Res* 1998; 39: 584, #3971.
19. Goillot E, Combaret V, Ladenstein R, *et al.* Tumor necrosis factor as an autocrine growth factor for neuroblastoma. *Cancer Res* 1992; 52: 3194–200.
20. Sinkovics JG, Horvath JC. Virological and immunological connotations of apoptotic and anti-apoptotic forces in neoplasia. *Int J Oncol* 2001; 19: 473–88.
